# Results of Surgical Reintervention After Suboptimal Initial Resection for Locoregional Neuroendocrine Tumors of the Small Intestine

**DOI:** 10.1002/wjs.12582

**Published:** 2025-04-16

**Authors:** Detlef K. Bartsch, Norman Krasser‐Gercke, Moritz Jesinghaus, Jannis Görlach, Frederike Eilsberger, Anja Rinke, Elisabeth Maurer

**Affiliations:** ^1^ Department of Visceral‐, Thoracic‐ and Vascular Surgery Philipps‐University Marburg Marburg Germany; ^2^ Institute of Pathology Philipps‐University Marburg Marburg Germany; ^3^ Department of Diagnostic and Interventional Radiology Philipps‐University Marburg Marburg Germany; ^4^ Department of Nuclear Medicine Philipps‐University Marburg Marburg Germany; ^5^ Department of Gastroenterology and Endocrinology Philipps‐University Marburg Marburg Germany

**Keywords:** small intestine neuroendocrine tumor, suboptimal initial resection, surgical reintervention

## Abstract

**Background:**

Complete resection is the only chance for cure in small intestine neuroendocrine neoplasms (SI‐NEN). Previous ENETS guidelines proposed standards for the surgery of SI‐NEN, which should be followed to provide long‐term disease‐free survival.

**Aim:**

To analyze the results of reintervention for locoregional SI‐NEN (stages I–III) after suboptimal initial resection.

**Methods:**

Perioperative characteristics of all patients who underwent surgical reintervention after suboptimal initial resection (SIR) of locoregional SI‐NEN were retrieved from a prospective database. Patient characteristics, initial and redo procedures, imaging before reintervention, pathological results of SIR, and after reintervention, including missed primary tumors and lymph node metastases, were retrospectively analyzed.

**Results:**

During a 15 years period, 21 of 93 (22%) patients had surgical reinterventions after SIR. In 20 of 21 (95%) cases, the initial resection was performed outside an ENETS center of excellence. Ten (48%) of those cases were emergency operations because of the bowel obstruction or bowel bleeding. Seven SIR (33%) cases were performed laparoscopically, and in another 5 (24%) cases, a complete endoscopic mucosa resection was performed. Imaging before reintervention visualized residual disease in 15 of 21 (71%) patients. Surgical reintervention included either lymphadenectomy alone (LAD, *n* = 3) or small bowel resection plus systematic LAD (*n* = 12) or right hemicolectomy/ileocecal resection with systematic LAD (*n* = 6), respectively. In 19 of 21 (90%) patients, a R0 resection could be achieved. One patient (5%) experienced postoperative clinically relevant complications. According to pathology, in 10 (48%) patients lymph node metastases, in 6 (29%) patients additional primary tumors, and in 5 (24%) patients, both lymph nodes metastases and primary tumors were left behind in the SIR. After mean follow‐up of 52 months, 16 (76%) of 21 patients were free of disease, 4 (19%) patients were alive with disease, and 1 patient deceased of an unrelated cause.

**Conclusion:**

The proposed standards to resect locoregional SI‐NEN should be followed to avoid SIR, although the prognosis after adequate surgical reintervention is good.

## Graphical abstract

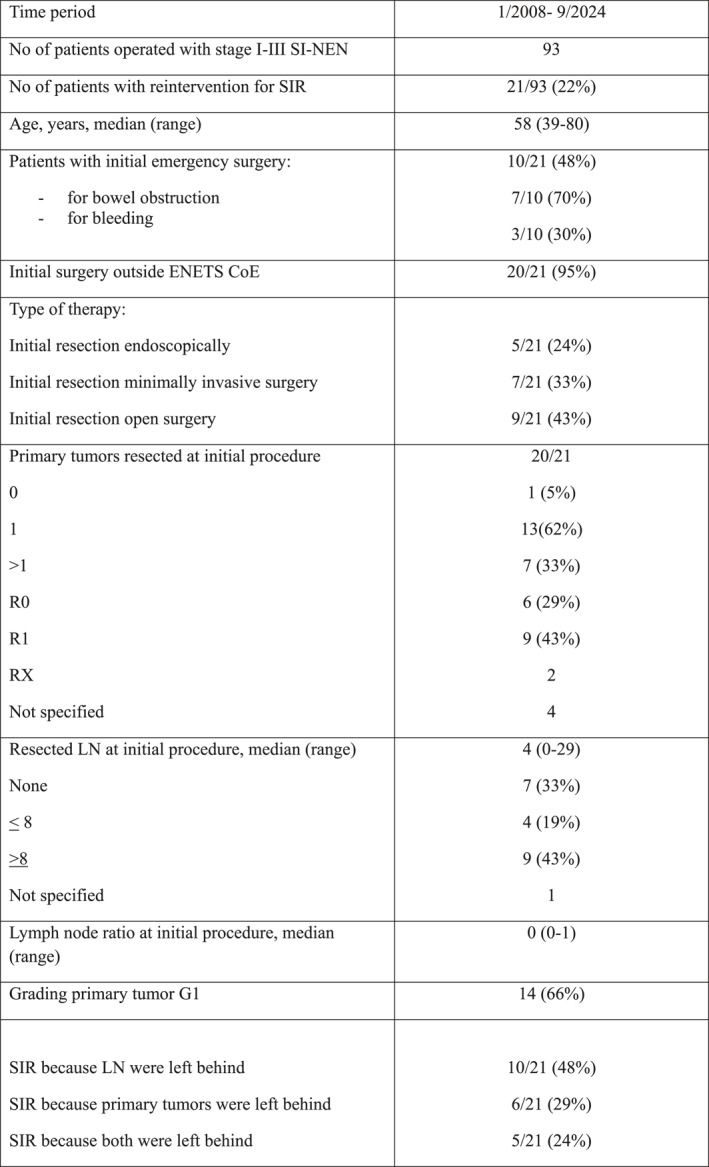

## 1 Introduction

Small intestinal neuroendocrine neoplasms (SI‐NEN) are the most common small bowel tumors, with a current incidence of 0.67–0.81/100,000 individuals/year [1, 2, 3]. SI‐NEN typically present as small primary tumor(s) (< 20 mm), are in the ileum, occur multiple in about 30% of patients, and are associated with lymph node metastases in 80%–90% and liver metastases in 60%–70% at the time of diagnosis [4, 5, 6]. Mesenteric lymph node metastases often grow larger than the primary tumor, and together with typical peritumoral fibrosis, they can cause intestinal obstruction or bowel ischemia [7, 8]. However, lymph node metastases can also be microscopic and not visible on preoperative functional imaging [9]. Complete surgical resection is the only chance for cure. Given the potential to metastasize, all SI‐NEN, even smaller as 10 mm, can be considered aggressive disease and should thus be radically resected whenever possible [10]. ENETS guidelines [10, 11] recommend surgical standards for the curative resection of localized SI‐NEN, which include a vessel and intestine‐sparing systematic lymphadenectomy (at least 8 lymph nodes) irrespective of visible lymphadenopathy or mesenteric involvement. Bidigital palpation of the entire small to detect multiple primary tumors should be a routine before deciding on the type of bowel procedure. These recommendations hold even true when SI‐NEN are small and incidentally discovered during screening ileocolonoscopy [11, 12]. Following these rules, one can achieve an excellent prognosis for locoregional (stages I–III) SI‐NENs with a disease‐free survival of approximately 95% for stages I and II and 72%–95% for stage III tumors [13, 14]. Several retrospective studies have demonstrated that a significant number of initial resections for locoregional SI‐NEN do not follow these recommended surgical standards. Twelve to 20% of the patients received no lymphadenectomy [5, 12, 15, 16]. SI‐NEN surgery is sometimes an emergency intervention, frequently performed by nonspecialized surgeons unaware of their management specificities and sometimes even unaware of SI‐NEN presence [17]. A French retrospective multicenter study proposed that complete primary surgery with appropriate lymphadenectomy was not performed in 50% of the operated patients [18]. Some patients were also treated with endoscopic resections for incidentally detected small SI‐NEN during ileocolonoscopy, although this is not an adequate treatment according to guidelines [10, 11, 19]. The French guideline recommends discussing surgical reintervention after SIR in a multidisciplinary tumor board [20]. Since the indications for reintervention and their outcomes after suboptimal initial resection (SIR) are yet not well defined, these were evaluated by the present study.

## 2 Material and Methods

The prospective surgical database of the ENETS Center of Excellence of the Philipps‐University Marburg was searched to identify patients with a histologically confirmed locoregional (stages I–III) SI‐NEN who underwent surgical reinterventions within 6 months after SIR between January 2008 and September 2024. Patients with liver and/or unresectable lymph node metastases at the mesenteric root (level IV according to Ohrvall et al. [21] and level III according to Bartsch et al. [22]) and patients with duodenal NENs were excluded. SIR could have been endoscopic or surgical. The initial procedure was considered as SIR based on operative and pathological reports and postoperative functional somatostatin receptor imaging, if at least one of the following criteria was fulfilled: (a) no palpation of the entire small intestine, (b) less than 8 lymph nodes removed, and (c) suspected residual locoregional tumor on postoperative imaging. In case of suspected SIR, multidisciplinary tumor board participants generally recommended surgical reintervention, if the patient was in a good general condition.

All patient data were pseudonymized and retrospectively analyzed. Pathology reports and operative notes of initial resections and surgical reinterventions were rereviewed regarding the location and size of the primary tumor(s), presence of mesenteric fibrosis, type of bowel resection, and the extent of lymphadenectomy. An emergency procedure was defined as an unplanned surgery for SI‐NEN‐related bowel obstruction or bleeding within 24 h of hospital admission.

Histopathological parameters included size of primary tumor(s), primary tumor multiplicity, number of resected lymph nodes, and metastatic lymph nodes and tumor grading assessed using the Ki‐67 proliferation index [23].

Morphological imaging with computed tomography (CT) and/or magnetic resonance imaging (MRI) as well as metabolic imaging with 68gallium‐labeled somatostatin analogs (68Ga‐DOTA–PET/CT) before reintervention was reassessed by experienced radiology (JG) and nuclear medicine (FE) physicians with regard to visible residual disease. Visible residual lymph node metastases were categorized as easily resectable (level I) or potentially difficult to resect (stage II) according to the Marburg classification [22].

The standard surgical reintervention approach was bidigital palpation of the entire small bowel in order to detect multifocal tumors by two surgeons prior to any resection. In case of residual primary SI‐NEN(s), either organ‐sparing small bowel resection or if the tumor(s) were located within the distal 30 cm of the ileum, a right hemicolectomy or ileocecal resection was performed. This was always combined with a systematic mesenteric lymphadenectomy up to the level of the inferior border of the pancreas body (level III according to Ohrvall [21]), independent of the presence of lymph node metastases. In case of residual lymph node metastases only, vessel‐sparing systematic lymphadenectomy was performed.

Postoperative clinically relevant complications ≥ 3 according to Dindo–Clavien were considered for analysis [24]. Complete resection was determined based on the surgical report, the histopathological analysis stating a R0 resection, and the first postoperative imaging after 3–6 months confirming no evidence of disease.

Patients were followed until the evaluation date September 30th, 2024 or death at the departments of Visceral‐, Thoracic‐ and Vascular Surgery or Gastroenterology and Endocrinology of the Marburg University hospital. Follow‐up investigations included a physical examination, measurement of serum chromogranin A, abdominal cross‐sectional imaging (preferably MRI), and conceivably functional somatostatin receptor imaging. Follow‐up examinations took place every 6 months for 5 years and yearly thereafter according to ENETS guidelines [11, 25]. SI‐NEN recurrence was defined as either bowel recurrence, lymph node, or distant metastases confirmed using the functional somatostatin receptor imaging and/or histology. Selected data of some patients were part of a previous publication [26].

### 2.1 Statistical Analysis

Results were presented as frequencies with percentages for categorical variables and median with range for continuous variables as medians (interquartile ranges [IQRs]). In patients without residual tumor after reintervention, disease‐free survival (DFS), defined as the time from the reintervention date to recurrence or any cause of death, was determined using the Kaplan–Meier method. Statistical significance was defined as *p* < 0.05 for all analyses. Data analyses were computed with R Studio (v.1.1.456; RStudio Inc., Boston, MA).

## 3 Results

Twenty‐one of 93 (22.5%) patients underwent surgical reintervention for locoregional SI‐NEN during a 15 years period. Their median age at reintervention was 58 (range 39–80) years and eight patients (38%) were women. The median time from SIR to reintervention was 3 months (range 1–6) months and the median follow‐up was 57 (4–190) months after SIR, respectively, 52 (1–187) months after surgical reintervention.

Before SIR, 7 (33%) SI‐NENs were incidentally discovered during either ileocolonoscopy (*n* = 5) or during surgery for another reasons (*n* = 2). Thirteen (62%) patients had SI‐NEN‐related symptoms, most frequently abdominal pain, which led to emergency surgery in 10 (48%) patients. In 7 of 16 (44%) surgical patients, a bidigital palpation of the entire small bowel was performed. In one patient (5%), no primary tumor was resected, in 13 (62%) patients, one primary tumor was resected, and in 2 patients, more than one primary tumor was removed. Seven (33%) patients had none and 4 (19%) patients had less than 8 lymph nodes removed. Among the 16 patients with surgical SIR, 7 (33%) had laparoscopic resections. In six of these 7 patients, no complete palpation of the small bowel was performed, less than 8 lymph nodes were removed in 4 patients, and in only one of these 7 laparoscopically treated patients, multiple primary tumors were identified. Two of the 16 patients had further operations before reintervention because of postoperative complications, such as fascia dehiscence and/or bowel obstruction, due to an internal hernia.

In five (24%) patients, an endoscopic resection of a primary tumor was performed during ileocolonoscopy.

Twenty of the 21 (95%) patients had their initial rection outside an ENETS CoE (European Neuroendocrine Tumor Society Center of Excellence). The clinical characteristics of patients who underwent SIR are summarized in Table 1.

**TABLE 1:** Characteristics of patients with locoregional SI‐NEN and SIR at initial procedure.

Time period	1/2008–9/2024
No of patients operated with stages I–III SI‐NEN	93
No of patients with reintervention for SIR	21/93 (22%)
Age, years, median (range)	58 (39–80)
Female gender	8/21 (38%)
Patients with initial emergency surgery:	10/21 (48%)
For bowel obstruction	7/10 (70%)
For bleeding	3/10 (30%)
Initial surgery outside ENETS CoE	20/21 (95%)
Type of therapy:	
Initial resection endoscopically	5/21 (24%)
Initial resection minimally invasive surgery	7/21 (33%)
Initial resection open surgery	9/21 (43%)
Primary tumors resected at initial procedure	20/21
0	1 (5%)
1	13 (62%)
>1	7 (33%)
R0	6 (29%)
R1	9 (43%)
RX	2
Not specified	4
Resected LN at initial procedure, median (range)	4 (0–29)
None	7 (33%)
≤ 8	4 (19%)
> 8	9 (43%)
Not specified	1
Lymph node ratio at initial procedure, median (range)	0 (0–1)
Grading primary tumor G1	14 (66%)
Reasons for SIR	
SIR because LN were left behind	10/21 (48%)
SIR because primary tumors were left behind	6/21 (29%)
SIR because both were left behind	5/21 (24%)

Abbreviations: ENETS CoE, European Neuroendocrine Tumor Society Center of Excellence; LN, lymph nodes; SIR, suboptimal initial resection.

All 21 patients had post‐SIR imaging with functioning SRS imaging and four patients had additionally either MRI or CT cross‐sectional imaging, thus all patients had undergone imaging with at least one of the techniques before surgical reintervention. Residual tumor was suspected in 15 of 21 (72%) patients based on interval imaging. Residual primary tumor alone, LN metastases alone, or both primary tumor and LN metastases were suspected in 6, 10, and 5 patients, respectively. Urine 5‐HIAA was measured in all patients (mean 27, 6 μmol/L, and SD 22,9 [9.4–77]).

All 21 patients with SIR underwent surgical reintervention, including lymphadenectomy alone (*n* = 3), small bowel resection with systematic lymphadenectomy (*n* = 12), oncologic right hemicolectomy (*n* = 2), or ileocecal resection with systematic lymphadenectomy, along the superior mesenteric artery up the pancreatic body (*n* = 4), respectively (Table 2). In 19 (90%) patients, a R0 resection could be achieved according to the surgeon’s judgment. One patient (5%) had a postoperative clinically significant complication due to a segmental pulmonary artery embolism.

**TABLE 2:** Operative and pathological characteristics of surgical reinterventions after SIR.

Time to reoperation, months, median (range)	3 (1–6)
Indication for reintervention	
No of lymphadenectomy performed	7/21 (33%)
R1/R2 resection according to pathology	8/21 (39%)
Residual disease on imaging	15/21 (72%)
Residual disease on PET/CT	11/21 (52%)
Residual imaging on CT, MRI, or ultrasound	4/21 (19%)
Reoperations performed	
Only lymphadenectomy	3/21 (14%)
Small bowel resection plus lymphadenectomy	12/21 (57%)
Right hemicolectomy	2/21 (10%)
Ileocecal resection	4/21 (19%)
Additional primary tumors resected	11/21 (52%)
Only primary tumors	6/11
Both primary tumors and lymph node metastases	5/11
Additional lymph node metastases resected	10/21 (48%)
No. of resected primary tumors, median (range)	1 (1–8)
No. of lymph nodes resected, median (range)	13 (1/29)
No. of resected affected lymph nodes per patient, median (range)	3 (0–12)
R0‐resection achieved	19/21 (90%)
Patients with clinically relevant postoperative complications (CD ≥ 3)	1 (5%)
Patients with postoperative short bowel syndrome	0

Abbreviations: CD, Clavien–Dindo; No., number; SIR, suboptimal initial resection.

All 21 patients had residual disease based on histopathological analysis after reintervention (Table 2). Residual primary tumors were detected in 11 (48%) patients, in 4 of 7 patients after laparoscopic SIR. Ten (48%) patients showed lymph node metastases alone and 5 (24%) patients both primary tumor and lymph node metastases, whereas in the remaining 6 (29%) patients, only residual primary tumors were detected upon histopathology. A median of 13 (1–29) lymph nodes were removed and median 3 (0–12) affected lymph nodes were resected per patient. In 19 (90%) patients, a R0‐resection was accomplished based on histopathology. Among the 6 patients without imageable residual disease after SIR, 4 had lymph node metastases and 2 had residual primary tumor(s).

After a median follow‐up after reintervention of 52 (range 1–187) months, 16 of 19 (84%) patients with a complete tumor clearance due to surgical reintervention remained disease‐free (Table 3). The median disease‐free survival of these patients was 18 (IQR 9–51) months. Two patients developed tumor recurrence 6 and 8 months after re‐resection and are alive 47 and 86 months after surgical treatment. One disease‐free patient deceased of an unrelated cause after 42 months. The two patients with incomplete tumor resection are alive with 140 and 131 months after reintervention. All patients with persistent and recurrent disease underwent additional treatments, such as somatostatin analogs, PRRT (peptide radio receptor therapy), or TAE (trans arterial embolization) (Table 3).

**TABLE 3:** Follow‐up of patients with reinterventions after SIR.

	Patients with SIR (*n* = 21)
Follow‐up after SIR median (range)	57 (4–190)
Follow‐up after reintervention median (range)	52 (1–187)
Disease status	
NED	16
AWD	4
DOD	0
DURC	1
Other treatments (e.g., SSA and PRRT)	4

Abbreviations: AWD, alive with disease; DOD, dead of disease; DURC, deaf of unrelated cause; NED, no evidence of disease; PRRT, peptide radio receptor therapy; SIR, suboptimal initial resection; SSA, somatostatin analogs.

## 4 Discussion

Data on reintervention after SIR in SI‐NEN are limited regarding the indication and the strategy for reintervention as well as morbidity rates and oncological outcomes. This is surprising, since the rate of SIR in SI‐NEN surgery is relevant. The Uppsala group previously reported that 20% of their 603 resected patients had surgical reinterventions, but no details concerning specific indications and outcomes were provided [27]. This number is confirmed by the present study and a French study [18]. A “reintervention concept” in SI‐NEN was first proposed as an expert opinion by Sutton et al. in 2003 [28]. In 2020, the French intergroup guidelines recommended to discuss surgical reintervention, if the first surgery was suboptimal, especially in an emergency setting, or when fewer than eight lymph nodes were harvested, or residual disease was visualized on postoperative metabolic imaging, respectively [20]. A recent multicentric retrospective French study was as yet the only one reporting the results of reintervention in SI‐NEN patients with SIR [18]. It demonstrated that reintervention achieved complete tumor clearance in 16/21 (76%) patients with low morbidity and a median disease‐free survival of 70 months. The present study confirms these data, since in 19 of 21 patients with SIR, a R0 resection could be achieved by reintervention with a low rate (4.8%) of clinically relevant postoperative complications. Even more important, 16 of these 19 patients remained disease‐free after a median follow‐up of 52 months. The presented results support the hypothesis, that a reintervention after SIR can fully meet guideline recommendations [10, 11], if performed by a NET‐specialized surgeon. In the present and the French series [18], additional primaries could be identified and resected in up to 57% of patients and a median of 13 and 24 lymph nodes were (re)‐resected during reinterventions.

There are several reasons for SIR in SI‐NEN, unavoidable and avoidable. An unavoidable reason is life‐saving emergency surgery for either bowel obstruction (about 80%) and less frequently gastrointestinal bleeding, mesenteric ischemia, and intussusception [16, 18, 29]. The proportion of emergency procedures for SI‐NEN was 12%–31% in retrospective cohort studies [16, 17, 18, 27, 29, 30, 31] and 47% in the presented series. The emergency situation raises the risk for SIR, especially if the procedure has to be performed by nonspecialized surgeon unaware of the SI‐NEN management specificities. A recent unicentric retrospective North American study with 134 patients reported less resected lymph nodes (8 vs. 12) and a tendency toward a higher recurrence rate (42% vs. 31%) after emergency versus elective SI‐NEN surgery [17]. A retrospective study from Iceland with 113 patients reported that emergency surgery was associated with a 6‐fold risk of death in the first 12 months after surgery (HR: 5.99 and *p* = 0.01) and with more severe surgical complications [17]. A 2011 multivariate analysis of the French GTE database retained emergency surgery (OR 4.04 and 95% CI 2.01–8.11) as being significantly associated with relapse. However, an oncologic resection may not be possible or even contraindicated in the emergency context, for example, if a nonspecialized surgeon has to face gross mesenteric lymph node metastases [16]. In this case, the procedure should focus on the solution of the acute life‐threatening condition with the minimal possible procedure. Thereafter, the patient should be transferred to an experienced center to evaluate surgical reintervention by NET‐specialized surgeon [32, 33].

An avoidable reason for SIR is the omission of manual palpation of the entire small bowel before defining the extent and type of bowel resection as recommended in all guidelines [10, 11]. It has been shown that manual palpation of the entire small bowel maximizes the detection of multiple synchronous tumors, since these are missed in over 60% of patients on preoperative imaging [4, 32, 34, 35]. However, according to previous studies, the clinical practice is different, since in up to 50% of procedures, this requirement was not fulfilled. A recent French study has shown that the proposed standards for SI‐NEN surgery are better followed in high‐volume centers (>5 SI‐NEN surgeries/year) compared to low‐volume centers [36]. A bidigital palpation of the entire small bowel length (95.6% vs. 34.3% and *p* < 0.001) were more often performed in high volume centers, the number of patients with multiple SI‐NETs (96.3% vs. 65.1%), and as well as the number of tumors in those patients (median of 7 vs. 2 tumors) was significantly higher.

Another avoidable reason is the performance of a not adequate or even no lymphadenectomy, which has been the case in 52% of the present and 86% of the French study [18]. It has been demonstrated by several studies that adequate lymphadenectomy (> 8 lymph nodes resected) has a positive prognostic impact [12, 14, 37]. In a recent French study, the proportion of patients with at least 8 lymph nodes resected (43% vs. 25%) and a mesenteric mass resection (70% vs. 36%; *p* < 0.001) were significantly higher in high‐volume centers compared to low‐volume institutions [36]. Therefore, these authors recommended centralization of surgical SI‐NET care to provide optimal SI‐NET resection avoiding SIR.

A previous international survey reported that minimally invasive approaches for SI‐NEN resection have increasingly gained acceptance, especially in high‐volume centers, since 69% of 58 respondents from 20 countries applied minimally invasive surgery for the resection of SI‐NEN [38]. A recent analysis of the SEER‐database comparing patients with locoregional SI‐NEN who either underwent laparoscopic (*n* = 5, 298, 46.5%) or open resection (*n* = 6069, 53.5%) demonstrated similar results with regard to the number of resected lymph nodes and long‐term outcomes [39]. In a recent Dutch survey of 482 operated patients for SI‐NEN between 2005 and 2015, 29% of patients had a laparoscopic approach with a conversion rate of about 30% due to technical reasons [40]. The open resection group had significantly more multifocal tumors resected (24% vs. 14%), pN2 lymph nodes (18% vs. 7%), and stage IV disease (36% vs. 22%). The present study is the first that reports SIR after laparoscopic SI‐NEN resections. Of 7 patients with a laparoscopic resection in 2 patients, each mesenteric lymph node metastases and primary tumors were left behind and in 3 patients even both. A recent French study showed that the laparoscopic approach is more frequently used in low‐volume institutions, which have frequently not fulfilled the recommended standards [39]. From the authors perspective, the minimally invasive approach can be only adequately performed in the early stages of SI‐NENs without gross metastatic involvement of the mesenteric root, provided palpation of the entire small bowel is ensured. ENETS guidelines suggested a hybrid procedure combining laparoscopy and manual palpation of the entire small bowel after its exteriorization by a hand port [10].

Endoscopic resections are not recommended for SI‐NENs [11, 19]. However, five of our 21 patientshad initial endoscopic resections not biopsies. Since it has been shown that even SI‐NEN <10 mm size have already lymph node metastases [15] and that the presence of even micro metastases may have a negative prognostic impact [41], these lesions should be oncologically resected. One could argue that endoscopic resection of a small SI‐NÈN might be adequate, if postinterventional functioning SRS imaging is negative. It has to be noted that all five patients with endoscopic resection had postinterventional negative SRS imaging in the present study but showed lymph node metastases on histopathology. In the abovementioned French study, 1 of 3 patients with negative metabolic imaging after endoscopic resection had lymph node metastases [18]. Thus, the therapeutic strategy for such patients should be reintervention for thorough lymphadenectomy and entire palpation of the small intestine, unless major comorbidities contraindicate it.

The present study highlights the deficiency of morphological and functional imaging to detect residual disease, either primary tumors or lymph node metastases, after SIR. In the present study, about one third (6/21) patients had negative imaging before reintervention, although histological work‐up proved primary tumors and/or metastatic lymph nodes in the specimen. This supports the experience of Deguelte et al. who reported false negative imaging results in 20% of their patients before reintervention [18]. No prospective studies are available regarding the timing of follow‐up and the imaging modalities for patients with potentially curative SI‐NEN resection and none of the guidelines recommend routine functional imaging after the first “curative” surgery. Only in patients with advanced disease (loco‐regional LN involved and liver metastases), functioning SRS imaging is recommended after surgery within 3–6 months [11]. We believe that the routine use of functional SRI imaging should be indicated once within 3 months after all surgeries for SI‐NEN with curative intent to identify SIR by detection of any residual disease that could be potentially curative treated by surgical reintervention.

The limitation of the present study is its retrospective design and the unvalidated definition of SIR, the small number of reoperated patients with SIR, and the lack of a control group comprising not reoperated patients with SIR. The only way to exclude these drawbacks would be to conduct a prospective multicentric study, but such a study is rather unrealistic because of SI‐NEN rarity, its slow tumor progression, and also for ethical reasons.

In conclusion, the present study shows that SIR occur at least in 20% of patients with locoregional SI‐NEN. To detect SIR, postinterventional sensitive functional SRS imaging should be performed. SIR should be suspected, if there is a residual tumor on postoperative imaging, if no palpation of the entire small intestine was performed during surgery, and less than 8 lymph nodes were removed. Patients, who fulfill one of these criteria, should be assessed in tumor boards of expert NEN referral centers for surgical reintervention, since the results of reintervention after SIR by a NET‐specialist surgeon can provide long‐term disease‐free survival.

## Author Contributions

conceptualization: D.K.B. and E.M. data curation: E.M. and N.K.‐G. formal analysis: N.K.‐G. and E.M. investigation: A.R. and D.K.B. methodology: D.K.B. project administration: D.K.B. resources: A.R. software: N.K.‐G. supervision: D.K.B. validation: J.G., F.E. and M.J. visualization: J.G., F.E. writing – original draft: E.M. writing – review and editing: D.K.B.

## Consent

Informed consent was obtained from all subjects involved in the study.

## Conflicts of Interest

The authors declare no conflicts of interest.

## Institutional Review Board Statement

The study was conducted according to the guidelines of the Declaration of Helsinki and approved by the Ethics Committee of the Philipps‐University Marburg (Az. 25‐21 RS).

## Data Availability

The data presented in this study are available from the corresponding author upon request.

## References

[1] B. Niederle , U. F. Pape , F. Costa , et al., “Vienna Consensus Conference Participants. ENETS Consensus Guidelines Update for Neuroendocrine Neoplasms of the Jejunum and Ileum,” Neuroendocrinology 103, no. 2 (2016): 125–138, 10.1159/000443170. 26758972

[2] J. Eriksson , O. Norlén , M. Ögren , H. Garmo , C. Ihre‐Lundgren , and P. Hellman , “Primary Small Intestinal Neuroendocrine Tumors Are Highly Prevalent and Often Multiple Before Metastatic Disease Develops,” Scandinavian Journal of Surgery 110, no. 1 (2021): 44–50, 10.1177/1457496919874484. 31587594

[3] A. Dasari , C. Shen , D. Halperin , et al., “Trends in the Incidence, Prevalence, and Survival Outcomes in Patients With Neuroendocrine Tumors in the United States,” JAMA Oncology 3, no. 10 (2017): 1335–1342, 10.1001/jamaoncol.2017.0589. 28448665 PMC5824320

[4] K. J. Keck , J. E. Maxwell , A. F. Utria , et al., “The Distal Predilection of Small Bowel Neuroendocrine Tumors,” Annals of Surgical Oncology 25, no. 11 (2018): 3207–3213, 10.1245/s10434-018-6676-2. 30054825 PMC6525566

[5] B. M. Motz , P. D. Lorimer , D. Boselli , J. S. Hill , and J. C. Salo , “Optimal Lymphadenectomy in Small Bowel Neuroendocrine Tumors: Analysis of the NCDB,” Journal of Gastrointestinal Surgery 22, no. 1 (2018): 117–123, 10.1007/s11605-017-3524-9. 28819895

[6] E. Bertani , M. Falconi , C. Grana , et al., “Small Intestinal Neuroendocrine Tumors With Liver Metastases and Resection of the Primary: Prognostic Factors for Decision Making,” International Journal of Surgery 20 (2015): 58–64, 10.1016/j.ijsu.2015.06.019. 26074290

[7] O. Norlén , P. Stålberg , K. Öberg , et al., “Long‐term Results of Surgery for Small Intestinal Neuroendocrine Tumors at a Tertiary Referral Center,” World Journal of Surgery 36, no. 6 (2012): 1419–1431, 10.1007/s00268-011-1296-z. 21984144

[8] J. C. Yao , M. Hassan , A. Phan , et al., “One Hundred Years After Carcinoid: Epidemiology of and Prognostic Factors for Neuroendocrine Tumors in 35,825 Cases in the United States,” Journal of Clinical Oncology 26, no. 18 (2008): 3063–3072, 10.1200/jco.2007.15.4377. 18565894

[9] P. Addeo , G. Poncet , B. Goichot , et al., “The Added Diagnostic Value of ^18^F‐Fluorodihydroxyphenylalanine PET/CT in the Preoperative Work‐Up of Small Bowel Neuroendocrine Tumors,” Journal of Gastrointestinal Surgery 22, no. 4 (2018): 722–730, 10.1007/s11605-017-3645-1. 29235002

[10] S. Partelli , D. K. Bartsch , J. Capdevila , et al., and Antibes Consensus Conference participants , “ENETS Consensus Guidelines for Standard of Care in Neuroendocrine Tumours: Surgery for Small Intestinal and Pancreatic Neuroendocrine Tumours,” Neuroendocrinology 105, no. 3 (2017): 255–265, 10.1159/000464292. 28237989

[11] A. Lamarca , D. K. Bartsch , M. Caplin , et al., “European Neuroendocrine Tumor Society (ENETS) 2024 Guidance Paper for the Management of Well‐Differentiated Small Intestine Neuroendocrine Tumours,” Journal of Neuroendocrinology 36, no. 9 (2024): e13423, 10.1111/jne.13423. 38977327

[12] C. S. Landry , H. Y. Lin , A. Phan , et al., “Resection of At‐Risk Mesenteric Lymph Nodes Is Associated With Improved Survival in Patients With Small Bowel Neuroendocrine Tumors,” World Journal of Surgery 37, no. 7 (2013): 1695–1700, 10.1007/s00268-013-1918-8. 23657749 PMC3881007

[13] N. Habbe , V. Fendrich , A. Heverhagen , A. Ramaswamy , and D. K. Bartsch , “Outcome of Surgery for Ileojejunal Neuroendocrine Tumors,” Surgery Today 43, no. 10 (2013): 1168–1174, 10.1007/s00595-012-0408-1. 23143168

[14] F. M. Watzka , C. Fottner , M. Miederer , et al., “Surgical Treatment on NEN of Small Bowel: Retrospective Analysis,” World Journal of Surgery 40, no. 3 (2016): 749–758, 10.1007/s00268-016-3432-2. 26822157

[15] J. C. Walsh , D. F. Schaeffer , R. Kirsch , et al., “Ileal Carcinoid Tumors‐Small Size Belies Deadly Intent: High Rate of Nodal Metastasis in Tumors ≤ 1 cm in Size,” Human Pathology 56 (2016): 123–127, 10.1016/j.humpath.2016.05.023. 27327193

[16] C. Le Roux , C. Lombard‐Bohas , C. Delmas , et al., and Groupe d’étude des Tumeurs Endocrines (GTE) , “Relapse Factors for Ileal Neuroendocrine Tumours After Curative Surgery: A Retrospective French Multicentre Study,” Digestive and Liver Disease 43, no. 10 (2011): 828–833, 10.1016/j.dld.2011.04.021. 21641888

[17] N. Manguso , A. Gangi , N. Nissen , et al., “Long‐Term Outcomes After Elective *Versus* Emergency Surgery for Small Bowel Neuroendocrine Tumors,” American Surgeon 84, no. 10 (2018): 1570–1574, 10.1177/000313481808401006. 30747671

[18] S. Deguelte , C. Hammoutene , G. Poncet , et al., “Concept of Reintervention With Thorough Lymphadenectomy After Suboptimal Resection of Small‐Intestine Neuroendocrine Neoplasms: A Multicentre Preliminary Study,” Journal of Neuroendocrinology 34, no. 6 (2022): e13117, 10.1111/jne.13117. 35434838

[19] AuthorsCollaborators:Deutsche Gesellschaft für Gastroenterologie, Verdauungs‐ und Stoffwechselkrankheiten DGVSNetzwerk Neuroendokrine Tumoren NeT e.V. PatientenvertretungBundesorganisation Selbsthilfe NeuroEndokrine Tumoren e.V. NET‐sgh PatientenvertretungDeutsche Gesellschaft für Hämatologie und Medizinische Onkologie e.V. DGHO, und Arbeitsgemeinschaft Internistische Onkologie AIO der Deutschen Krebsgesellschaft e.VDeutsche Gesellschaft für Allgemein‐ und Viszeralchirurgie e.V. DGAVDeutsche Gesellschaft für Chirurgie DGCHDeutsche Gesellschaft für Endoskopie und Bildgebende Verfahren DGEBVDeutsche Gesellschaft für Nuklearmedizin e.V. DGNMDeutsche Gesellschaft für Innere Medizin DGIMDeutsche Gesellschaft für Endokrinologie DGEDeutsche Gesellschaft für Palliativmedizin e.V. DGPDeutsche Röntgengesellschaft e.V. DRGDeutsche Gesellschaft für Pathologie e.V./Bundesverband Deutscher Pathologen DGP/BDPDeutsche Gesellschaft für interventionelle Radiologie DGiR , “S2k‐Leitlinie Neuroendokrine Tumore [Practice Guideline Neuroendocrine Tumors ‐ AWMF‐Reg. 021‐27],” Zeitschrift für Gastroenterologie 56, no. 6 (2018): 583–681, 10.1055/a-0604-2924. 29890561

[20] L. de Mestier , C. Lepage , E. Baudin , et al., “Digestive Neuroendocrine Neoplasms (NEN): French Intergroup Clinical Practice Guidelines for Diagnosis, Treatment and Follow‐Up (SNFGE, GTE, RENATEN, TENPATH, FFCD, GERCOR, UNICANCER, SFCD, SFED, SFRO, SFR),” Digestive and Liver Disease 52, no. 5 (2020): 473–492, 10.1016/j.dld.2020.02.011. 32234416

[21] U. Öhrvall , B. Eriksson , C. Juhlin , et al., “Method for Dissection of Mesenteric Metastases in Mid‐gut Carcinoid Tumors,” World Journal of Surgery 24, no. 11 (2000): 1402–1408, 10.1007/s002680010232. 11038214

[22] D. K. Bartsch , S. Windel , V. Kanngießer , et al., “Vessel‐Sparing Lymphadenectomy Should Be Performed in Small Intestine Neuroendocrine Neoplasms,” Cancers (Basel) 14, no. 15 (2022): 3610, 10.3390/cancers14153610. 35892869 PMC9332577

[23] Publication of WHO Classification of Tumours . 5th Edition, Volume 1: Digestive System Tumours – IARC, (WHO), accessed December 4, 2024. https://www.iarc.who.int/fr/news‐events/publication‐of‐who‐classification‐of‐tumours‐5th‐edition‐volume‐1‐digestive‐system‐tumours/.

[24] D. Dindo , N. Demartines , and P.‐A. Clavien , “Classification of Surgical Complications: A New Proposal With Evaluation in a Cohort of 6336 Patients and Results of a Survey,” Annals of Surgery 240, no. 2 (2004): 205–213, 10.1097/01.sla.0000133083.54934.ae. 15273542 PMC1360123

[25] J. D. Brierley , M. K. Gospodarowicz , and C. Wittekind , “Well‐Differentiated Neuroendocrine Tumours of the Gastrointestinal Tract,” in The TNM Classification of Malignant Tumours. 8th ed. (Wiley Blackwell, 2016), 96–104.

[26] E. Maurer , M. Heinzel‐Gutenbrunner , A. Rinke , et al., “Relevant Prognostic Factors in Patients With Stage IV Small Intestine Neuroendocrine Neoplasms,” Journal of Neuroendocrinology 34, no. 1 (2022): e13076, 10.1111/jne.13076. 34964186

[27] O. Norlén , P. Stålberg , K. Öberg , et al., “Long‐Term Results of Surgery for Small Intestinal Neuroendocrine Tumors at a Tertiary Referral Center,” World Journal of Surgery 36, no. 6 (2012): 1419–1431, 10.1007/s00268-011-1296-z. 21984144

[28] R. Sutton , H. E. Doran , E. M. I. Williams , et al., “Surgery for Midgut Carcinoid,” Endocrine‐Related Cancer 10, no. 4 (2003): 469–481, 10.1677/erc.0.0100469. 14713260

[29] A. Blažević , W. T. Zandee , G. J. H. Franssen , et al., “Mesenteric Fibrosis and Palliative Surgery in Small Intestinal Neuroendocrine Tumours,” Endocrine‐Related Cancer 25, no. 3 (2018): 245–254, 10.1530/erc-17-0282. 29255095

[30] M. Evers , A. Rinke , J. Rütz , A. Ramaswamy , E. Maurer , and D. K. Bartsch , “Prognostic Factors in Curative Resected Locoregional Small Intestine Neuroendocrine Neoplasms,” World Journal of Surgery 45, no. 4 (2021): 1109–1117, 10.1007/s00268-020-05884-6. 33416940

[31] S. Snorradottir , A. Asgeirsdottir , S. Rögnvaldsson , J. G. Jonasson , and E. S. Björnsson , “Incidence and Prognosis of Patients With Small Intestinal Neuroendocrine Tumors in a Population Based Nationwide Study,” Cancer Epidemiol 79 (2022): 102197. 35716441 10.1016/j.canep.2022.102197

[32] S. Deguelte , M. Perrier , C. Hammoutene , G. Cadiot , and R. Kianmanesh , “Surgery and Perioperative Management in Small Intestinal Neuroendocrine Tumors,” Journal of Clinical Medicine 9, no. 7 (2020): 2319, 10.3390/jcm9072319. 32708330 PMC7408509

[33] E. Maurer and D. K. Bartsch , “Lokale Resektion Von Neuroendokrinen Tumoren Des Dünndarms (SI‐NEN): Aktuelle Prinzipien [Local Resection of Small Intestine Neuroendocrine Neoplasms (SI‐NEN): Current Principles],” Chirurgie 95, no. 10 (2024): 818–824, 10.1007/s00104-024-02102-0. 38771340

[34] A. Pasquer , T. Walter , P. Rousset , et al., “Lymphadenectomy During Small Bowel Neuroendocrine Tumor Surgery: The Concept of Skip Metastases,” Annals of Surgical Oncology 23, no. Suppl 5 (2016): 804–808, 10.1245/s10434-016-5574-8. 27613554

[35] M. N. Figueiredo , L. Maggiori , S. Gaujoux , et al., “Surgery for Small‐Bowel Neuroendocrine Tumors: Is There Any Benefit of the Laparoscopic Approach?,” Surgical Endoscopy 28, no. 5 (2014): 1720–1726, 10.1007/s00464-013-3381-x. 24380996

[36] M. Kalifi , S. Deguelte , M. Faron , et al., “The Need for Centralization for Small Intestinal Neuroendocrine Tumor Surgery: A Cohort Study From the GTE‐Endocan‐RENATEN Network, the CentralChirSINET Study,” Annals of Surgical Oncology 30, no. 13 (2023): 8528–8541, 10.1245/s10434-023-14276-8. 37814184

[37] M. Y. Zaidi , A. G. Lopez‐Aguiar , M. Dillhoff , et al., “Prognostic Role of Lymph Node Positivity and Number of Lymph Nodes Needed for Accurately Staging Small‐Bowel Neuroendocrine Tumors,” JAMA Surgery 154, no. 2 (2019): 134–140, 10.1001/jamasurg.2018.3865. 30383112 PMC6439661

[38] E. Kaçmaz , A. F. Engelsman , W. A. Bemelman , et al., “International Survey on Opinions and Use of Minimally Invasive Surgery in Small Bowel Neuroendocrine Neoplasms,” European Journal of Surgical Oncology 48, no. 6 (2022): 1251–1257, 10.1016/j.ejso.2021.11.011. 34823919

[39] W. Wong , R. A. Perez Holguin , E. J. Olecki , et al., “Predictors and Outcomes of Minimally Invasive Surgery for Small Bowel Neuroendocrine Tumors: Minimally Invasive Surgery for SBNETs,” Journal of Gastrointestinal Surgery 26, no. 6 (2022): 1252–1265, 10.1007/s11605-022-05264-6. 35132564

[40] E. Kaçmaz , S. van Eeden , J. C. C. Koppes , et al., “Value of Laparoscopy for Resection of Small‐Bowel Neuroendocrine Neoplasms Including Central Mesenteric Lymphadenectomy,” Diseases of the Colon & Rectum 64, no. 10 (2021): 1240–1248, 10.1097/dcr.0000000000001915. 33661232

[41] G. Scaglione , P. Fransvea , E. Genco , and G. Rindi , “Isolated Tumor Cells Node Micro‐Metastasis in Early‐Stage Small Intestinal Neuroendocrine Tumor,” Endocrine Pathology 35, no. 3 (2024): 276–278, 10.1007/s12022-024-09823-2. 39172325

